# Efficient uptake and retention of iron oxide-based nanoparticles in HeLa cells leads to an effective intracellular delivery of doxorubicin

**DOI:** 10.1038/s41598-020-67207-y

**Published:** 2020-06-29

**Authors:** R. C. Popescu, D. Savu, I. Dorobantu, B. S. Vasile, H. Hosser, A. Boldeiu, M. Temelie, M. Straticiuc, D. A. Iancu, E. Andronescu, F. Wenz, F. A. Giordano, C. Herskind, M. R. Veldwijk

**Affiliations:** 1 “Horia Hulubei” National Institute for Research and Development in Physics and Nuclear Engineering, Department of Life and Environmental Physics, Reactorului 30, 077125 Magurele, Romania; 2 Politehnica University of Bucharest, Department of Science and Engineering of Oxide Materials and Nanomaterials, Polizu 1-7, 011061 Bucharest, Romania; 3 Heidelberg University, Medical Faculty Mannheim, Universitätsmedizin Mannheim, Department of Radiation Oncology, Theodor-Kutzer-Ufer 1-3, 68167 Mannheim, Germany; 4 Heidelberg University, Medical Faculty Mannheim, Universitätsmedizin Mannheim, Center for Biomedicine and Medical Technology, Department of Anatomy and Developmental Biology, Theodor-Kutzer-Ufer 1-3, 68167 Mannheim, Germany; 5 National Institute for Research and Development in Microtechnologies, Laboratory of Nanobiotechnology, Erou Iancu Nicolae 12A, 077190 Bucharest, Romania; 6 ”Horia Hulubei” National Institute for Research and Development in Physics and Nuclear Engineering, Department of Applied Nuclear Physics, Reactorului 30, 077125 Magurele, Romania; 7 University Medical Center Freiburg, Hugstetter Straße 55, 79106 Freiburg, Germany

**Keywords:** Nanobiotechnology, Cancer therapy, Drug development, Preclinical research, Nanomedicine, Chemical engineering

## Abstract

The purpose of this study was to construct and characterize iron oxide nanoparticles (IONP_CO_) for intracellular delivery of the anthracycline doxorubicin (DOX; IONP_DOX_) in order to induce tumor cell inactivation. More than 80% of the loaded drug was released from IONP_DOX_ within 24 h (100% at 70 h). Efficient internalization of IONP_DOX_ and IONP_CO_ in HeLa cells occurred through pino- and endocytosis, with both IONP accumulating in a perinuclear pattern. IONP_CO_ were biocompatible with maximum 27.9% ± 6.1% reduction in proliferation 96 h after treatment with up to 200 µg/mL IONP_CO_. Treatment with IONP_DOX_ resulted in a concentration- and time-dependent decrease in cell proliferation (IC_50_ = 27.5 ± 12.0 μg/mL after 96 h) and a reduced clonogenic survival (surviving fraction, SF = 0.56 ± 0.14; versus IONP_CO_ (SF = 1.07 ± 0.38)). Both IONP constructs were efficiently internalized and retained in the cells, and IONP_DOX_ efficiently delivered DOX resulting in increased cell death vs IONP_CO_.

## Introduction

Chemotherapy is an essential systemic component in modern multimodal cancer treatment, yet one of the main disadvantages of anticancer chemotherapeutics is toxicity to the normal tissue.

The use of nano-sized carriers as intracellular transporters for the active substances not only promises to reduce the total drug amount administered, while potentially improving the treatment’s efficiency by enhancing the local dose in the tumour, but also can help to improve the specificity and targeting of the active substance, thereby reducing the side-effects associated with chemotherapy^1^.

In nano-carriers, drugs can be transported to the tumour site through the enhanced permeability and retention effect^2–4^, magnetic targeting^5–8^ and protected until they find a triggering stimuli to release, like pH variations^9–12^, temperature^13,14^, radiation-induced release^15–19^.

The use of iron oxide nanoparticles in the construction of nano-systems for the delivery of chemotherapeutics not only enables active magnetic targeting to the tumour site, but also offers additional functions that make them suitable for diagnosis (contrast substance in MRI^20–22^) or enhanced anticancer activity using hyperthermia^23^.

Conjugation with other compounds can add to the multi-functionality of these nanomaterials and implement properties such as increased and/or specific^24–26^ internalization in cancer cells, but can also help to modulate the release of the active substance: (1) by protecting the drug, (2) by delaying the release or (3) by releasing the substance on demand (mediated by pH, heat, light, biological enzymes, etc.).

For these reasons, we designed and synthesized core-shell iron oxide (Fe_3_O_4_) nanoparticles functionalized with soft polyethylene glycol (PEG) layers (IONP_CO_) that entrap and protect the drug, the anthracycline doxorubicin (DOX) until delivery. DOX was chosen as a chemotherapeutic model due to its current clinical use in the management of various cancers, including cervical cancer, but also because of its traceability (fluorescence upon excitation). The novelty of this strategy comes from the synthesis method which is based on a room temperature adapted Massart approach combined with a post-synthesis encapsulation, resulting in core-shell- like PEG-conjugated highly crystalline Fe_3_O_4_ nanoparticles.

The aim of the study was to determine the uptake and retention efficacy of the IONP and to test their ability to induce cell death in HeLa cells by incorporation of DOX into the IONP_CO_. To the best of our knowledge, we evaluated for the first time the intracellular retaining and fate of PEG-coated iron oxide nanoparticles (qualitatively and also quantitatively) over a time range longer than one complete cell cycle, after the NP exposure had been discontinued. IONP_CO_ had the ability to encapsulate and deliver the chemotherapeutic doxorubicin directly into the intracellular cytoplasmic compartment, but were also retained until the death of the malignant cell.

## Results

### Physical and chemical characterization of the IONP

**Figure 1:**
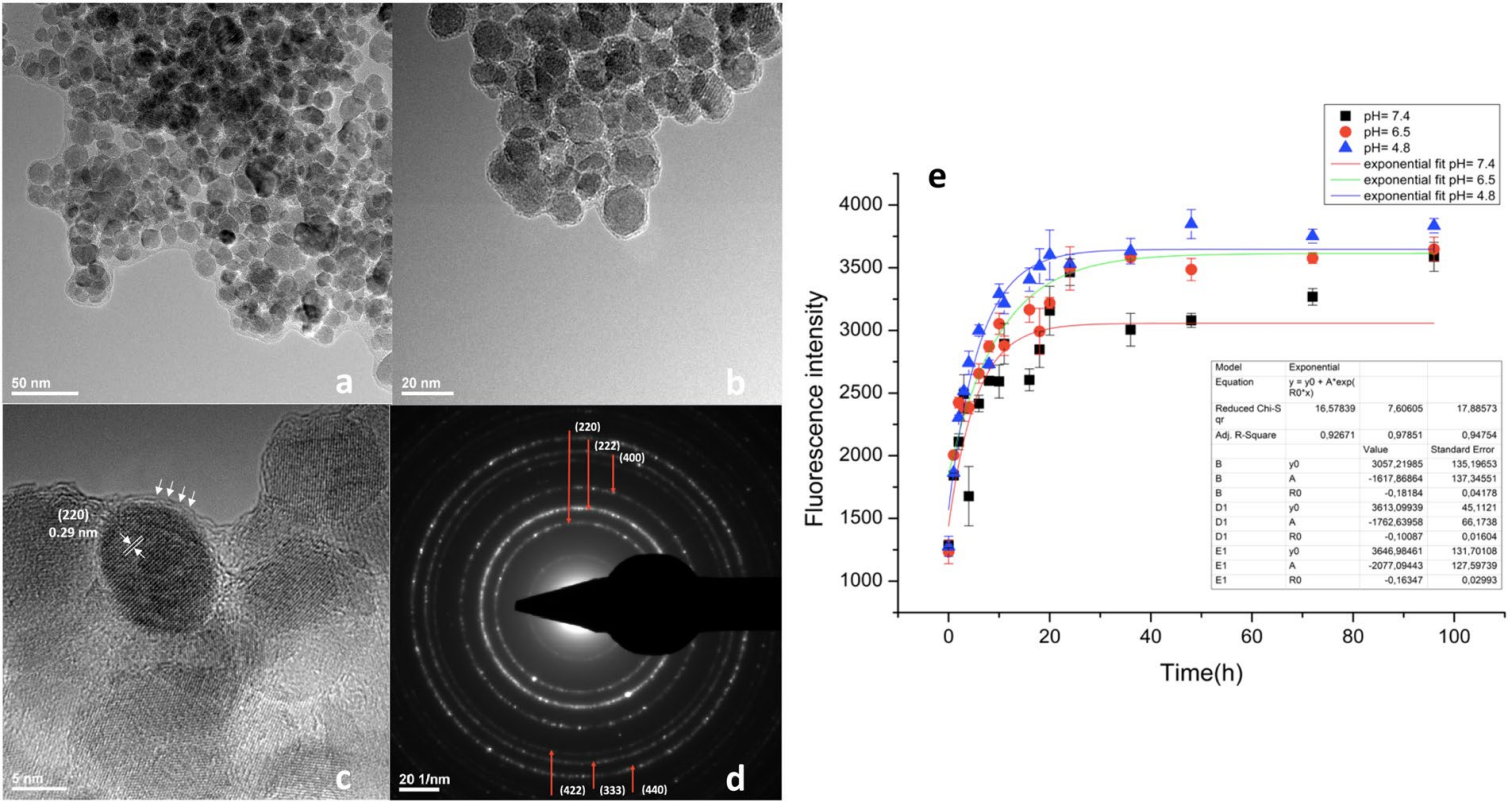
Structural and compositional characterization of IONP. (**a,b**) Transmission electron microscopy (TEM); (**c**) High resolution (HR)- TEM; (**d**) Selected area electron diffraction spectrum (SAED); (E) Delivery of doxorubicin from the IONP_DOX_ construct at 37 °C (0–96 h).

Synthesis of IONP using a three-step synthesis method leads to the generation of highly crystalline individual iron oxide cores with average diameters of 12.82 ± 2.73 nm (Fig. 1a–c), showing diffraction rings characteristic for face-centred spinel structured magnetite ((220), (222), (400), (422), (333), (440)) (Fig. 1d). Individually covered NPs with PEG were organized as core-shell-like nano-constructs, as shown by high resolution transmission electron microscopy (HR-TEM; Fig. 1c; PEG shell emphasized with white arrows). HR-TEM confirmed the data on crystallinity and emphasized the presence of the (220) plane of 0.29 nm width (Fig. 1c), characteristic for the magnetite phase. Conjugation with PEG also lead to a stabile dispersion of the NPs stock solution in water, a mean hydrodynamic diameter of 164.2 nm being measured (prior to ultrasound dispersion). Zeta potential measurements showed good stability (14.80 mV for stock solutions with no prior ultrasound treatment). Loading of the DOX resulted in an increase in the hydrodynamic diameter (369.1 nm mean diameter) and a change of surface charge to negative values (−20.9 mV zeta potential). Both of the constructs were monodisperse systems, as the values of the polydispersity index (PDI) were below 0.3 (0.233 for IONP_CO_ and 0.238 for IONP_DOX_). Quantitative determination of the DOX-loading content in IONP showed a value of 1.11 wt% (Supplementary Material Section 2).

### Release of DOX from IONP_DOX_

The release experiments were carried out in three biologically relevant culture media with different pH values: 7.4 pH, which is characteristic for physiologic plasma, 6.5 pH which is relevant for tumour microenvironment^27,28^ and 4.8 pH, which is encountered in the intra-lysosomal compartment^29^. IONP_DOX_ showed a rapid, initial release which was not significantly affected by the pH (Fig. 1e).

### Hemocompatibility of IONP

The hemolytic potential (Supplementary Table S2) was below 5% (ASTM standard E2524-08). Thus, neither IONP_CO_ nor IONP_DOX_ produced a hemolytic effect at the concentrations used.

### Internalizing and retention of IONP in HeLa cells

**Figure 2:**
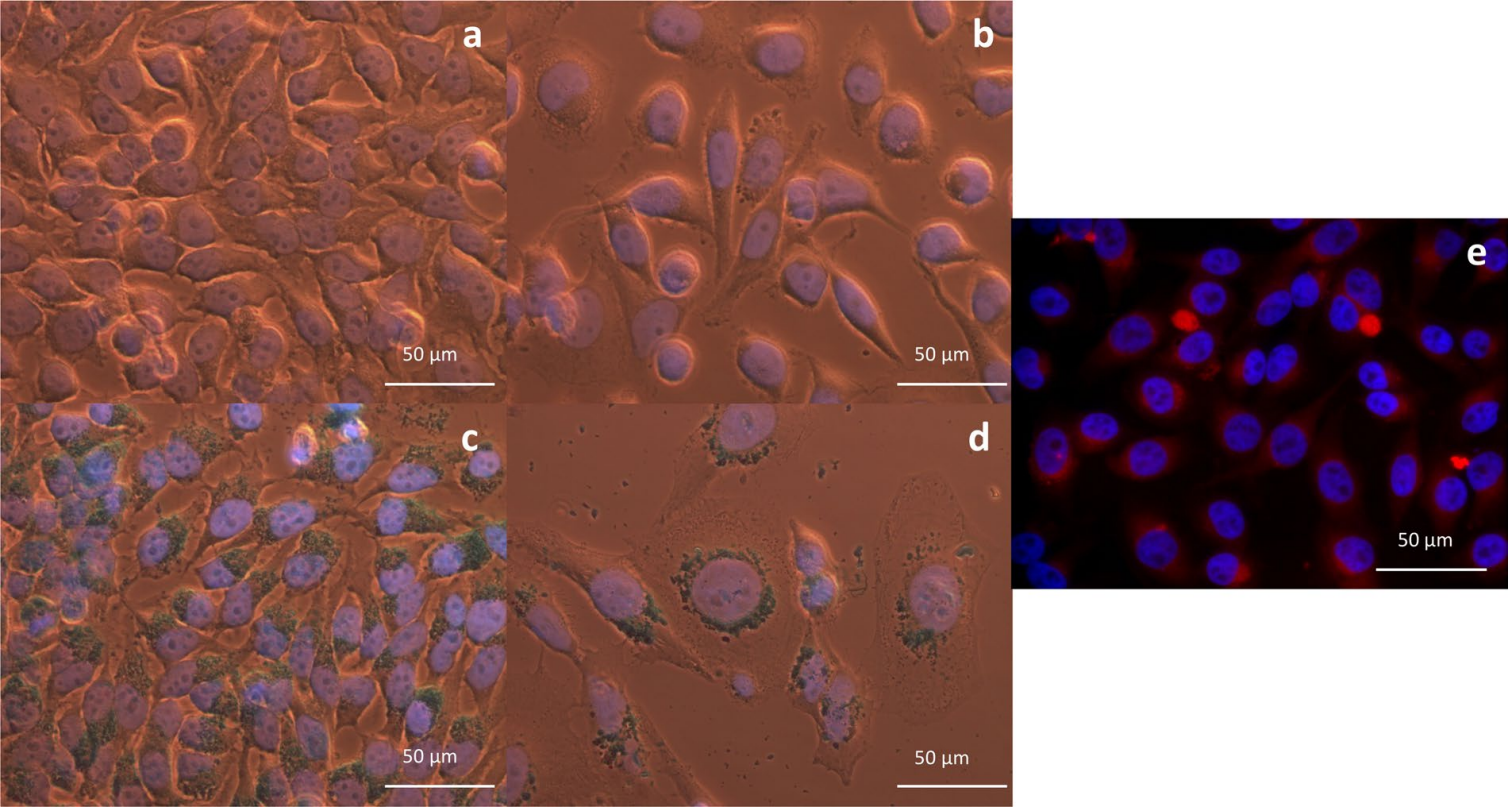
Morphological characterization of HeLa cells after IONP treatment. Bright-field images of HeLa cells incubated for 16 h with: (**a**) PBS; (**b**) DOX (1.11 μg/mL equivalent concentration to IONP_DOX_; Supplementary data Section 2); (**c**) IONP_CO_ (100 μg/mL) and (**d**) IONP_DOX_ (100 μg/mL); IONP are stained blue after Prussian Blue staining, 40x magnification (oil). Fluorescence images of HeLa cells 24 h after incubation for 16 h with: (**e**) 100 μg/mL or IONP_DOX_. Nuclei are stained with DAPI (blue), red staining is fluorescence from incorporated DOX; 40x magnification.

At 24 h after 16 h incubation with nanoparticles, internalized IONP were shown not to translocate into the nucleus, remaining organized as light blue-coloured agglomerates covering the outer nuclear membrane (Fig. 2c,d). The morphology of the samples was not affected in case of IONP_CO_-exposed HeLa cells (Fig. 2c). However, the cell density was affected by DOX from the IONP_DOX_ samples (Fig. 2d) and by equivalent amounts of free DOX (Fig. 2b). The morphology of IONP_DOX_-treated HeLa cells changed, becoming rounder and larger. In addition, nuclei increased in volume. By adjusting the focusing plane, different depth levels of accumulated IONP could be differentiated in the cell (Supplementary Figs. S1, S2). Thus, most nanoparticle agglomerates appeared in a perinuclear location in the cell. Still, some can be seen directly interacting with the cell membrane during the internalizing process.

In case of IONP_DOX_, localizing of the nano-constructs inside the cells was confirmed by fluorescence detection of DOX 24 h after 16 h incubation with DOX-loaded nanoparticles (Fig. 2e). A signature red fluorescence of the IONP_DOX_ aggregates (sub-micron spherical structures in the peri-nuclear area and the cytoplasm) was observed with a weaker intensity in the remaining cytoplasm and in the nucleus (Fig. 2e).

**Figure 3:**
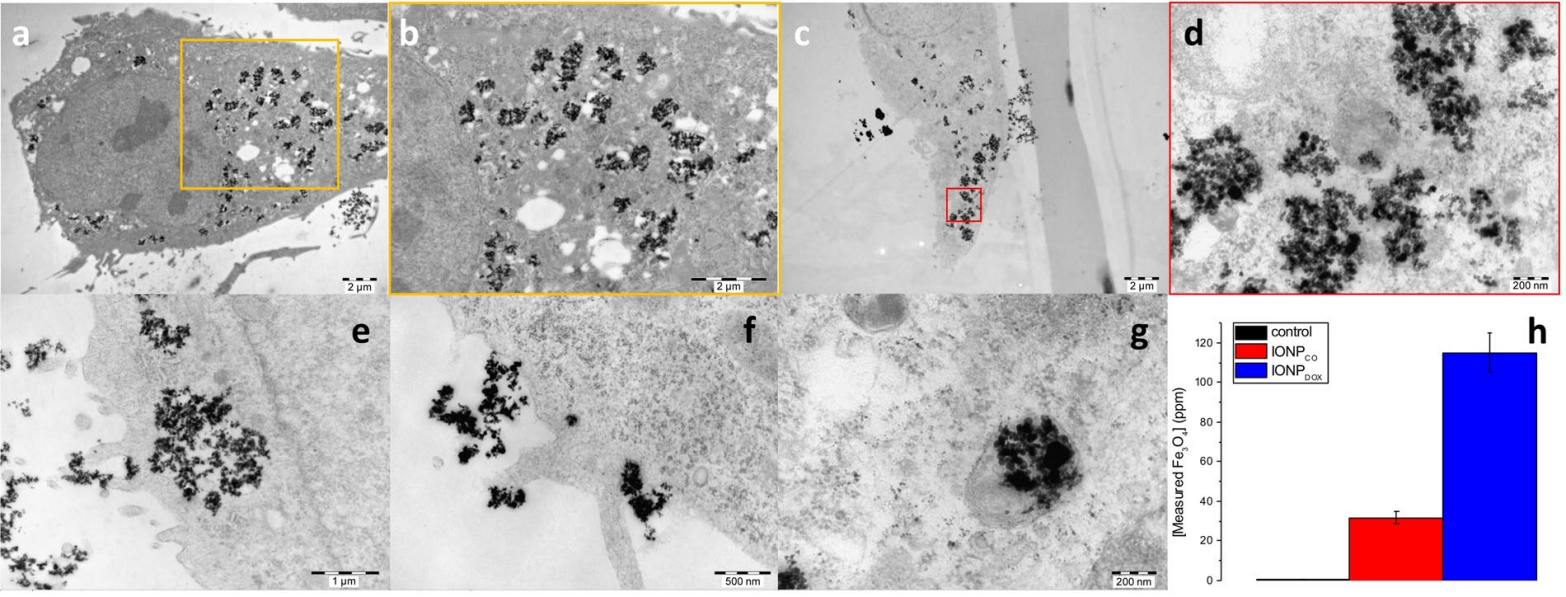
Internalization of IONP in HeLa cells. HeLa cells exposed to IONP_CO_ for 16 h: (**a**) overview of the cell, scale = 2 µm; (**b**) magnification of the area marked with yellow square in (**a**); HeLa cells exposed to IONP_DOX_ for 16 h: (**c**) overview of the whole cell, scale = 2 µm; (**d**) magnification of the area marked with red square in (**c**), scale = 200 nm. HeLa cells exposed to IONP for 16 h: (**e**) detail of macropinocytosis internalization of IONP, scale = 1 μm; (**f**) detail of endocytosis internalization of IONP, scale = 500 nm; (**g**) detail of lysosome entrapment of IONP, scale = 200 nm and **(h**) Quantity of internalized Fe_3_O_4_ in HeLa cells exposed to 0, respectively 100 ppm IONP during 16 h at 24 h after NP removal; data are presented as percentage of untreated control and are shown as mean ± SEM (n = 3).

Transmission electron images were acquired for HeLa cells exposed to different concentrations of IONP (100 and 500 μg/mL) for 16 h in order to show their internalization and localization in HeLa cells at 24 h after NP removal. This technique was also employed to potentially study the mechanisms of entrapment. Figure 3 emphasized the localizing of IONP as agglomerates in the perinuclear area and the cytoplasm. The results (Fig. 3e–g and Supplementary Fig. S4) show that both types of constructs are internalized via macropinocytosis (Fig. 3e) and sometimes smaller aggregates are internalized via endocytosis (Fig. 3f), eventually being transferred in lysosomes (Fig. 3g). At 24 h after the end of NP incubation period, the IONP_CO_ appeared to be entrapped in intracellular vesicles (Fig. 3a,b and, while IONP_DOX_ aggregates were localized in both vesicular structures and appeared free in cytoplasm (Fig. 3c,d).

Particle-induced X-Ray emission (PIXE) quantitative analysis of Fe_3_O_4_ interacting with HeLa cells yielded a concentration of 31.66 ± 3.06 pg Fe_3_O_4_ /cell in HeLa cells exposed to IONP_CO_, and 115.2 ± 9.8 pg Fe_3_O_4_ /cell for IONP_DOX_ (Fig. 3h).

### Effect of IONP on the proliferation kinetics of HeLa cells

The effect of IONP on the proliferation of HeLa cells was determined for a broad concentration range of IONP_CO_ and IONP_DOX_ (0–200 µg/mL) during 48–96 h incubation. Results were shown relative to controls (untreated HeLa cells = 100%).

**Figure 4:**
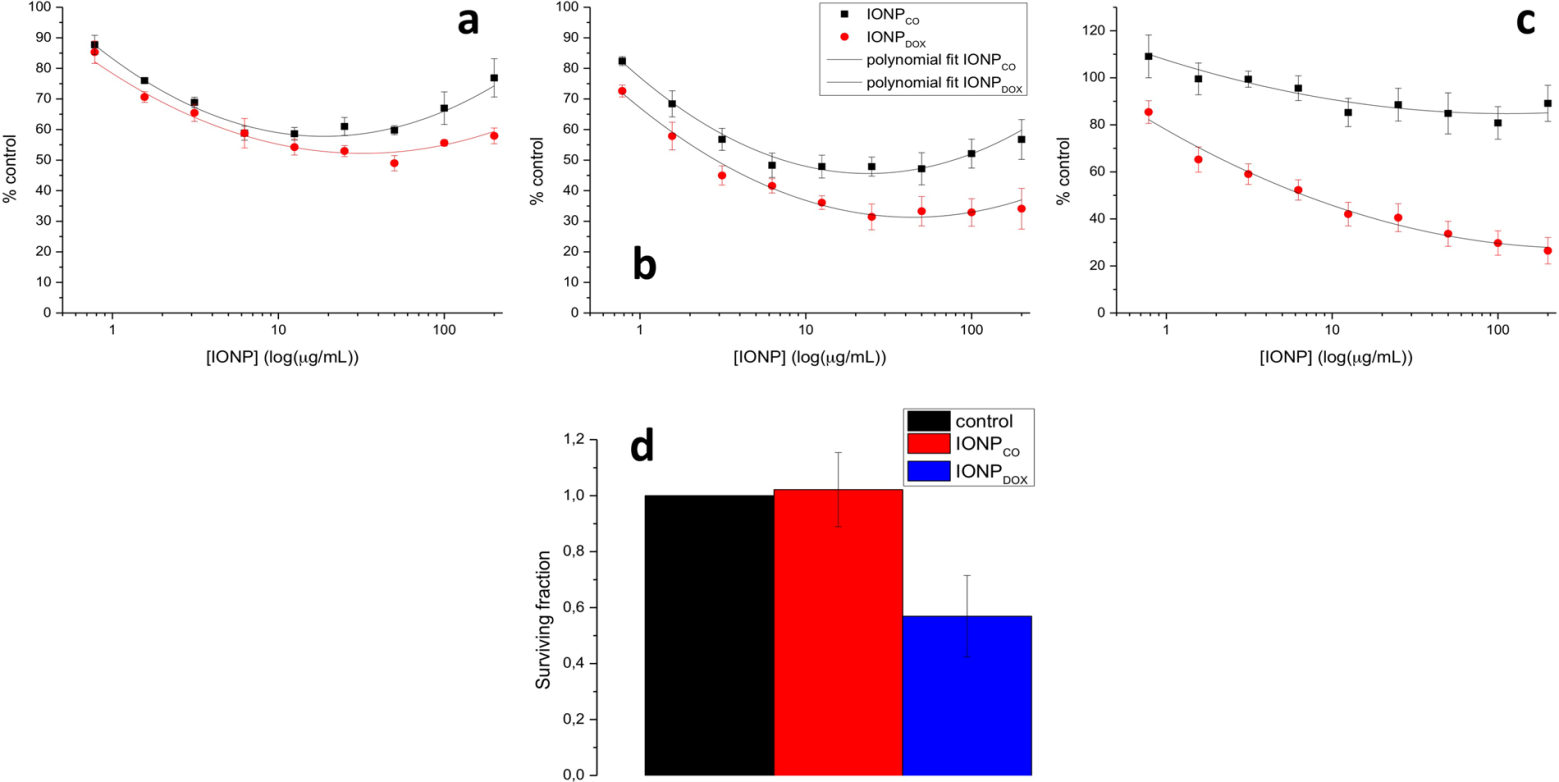
Cytotoxicity of IONP on HeLa cells. (**a–c**) Proliferation kinetics of HeLa cells incubated with IONP during 48, 72 and 96 h. One-way ANOVA statistical analysis revealed a significant difference between treated groups and control; Two-way ANOVA statistical analysis proved significant difference between IONPs and DOX-IONPs (P < 0.0001 for 48 h; P < 0.0001 for 72 h; P < 0.0001 for 96 h). Also, the presence of DOX in the construct induced a significant reduction of proliferation, compared to equivalent concentrations of IONP_CO_ (P = 0.0003 for 48 h; P < 0.0001 for 72 h; P < 0.0001 for 96 h). (**d**) Clonogenic survival of HeLa cells seeded in the colony formation assay after exposure to 100 μg/mL IONP for 16 h. Data are presented as percentage of untreated control and are shown as mean ± SEM (n = 3).

With IONP_CO_, a time and concentration-dependent decrease in proliferation was observed until 72 h of incubation with nanoparticles (Fig. 4a,b). All results were statistically significant relative to untreated cells (One-way ANOVA, p < 0.05). However, after 96 h of incubation, no significant changes in relative absorbance were observed compared to control samples, a maximum reduction of relative absorbance being 10.88 ± 7.68% for the highest concentration employed (200 µg/mL IONP) (Fig. 4c).

DOX-free nanoparticles caused a delay in cells proliferation up to 72 h. This effect was diminished at short incubation periods (24/48 h), the effect of IONP_CO_ on the HeLa cells could be rather correlated with an initial cytotoxic or growth inhibitory effect, as control cells normally underwent maximum 2 divisions during this time interval (doubling time of HeLa cells is ~18 h^30^). The effect on the cell's ability to reduce the tetrazolium salt might be justified by an initial toxicity with time (Fig. 4a,b). At later time points (96 h), showing values close to control cells suggested that the initial effect of the IONP was rather based on growth delay/inhibition than cell kill/cytotoxicity (Fig. 4c).

Incubation of HeLa cells with IONP_DOX_ showed a clear cytotoxic effect that increased with the concentration of administered NP and time (Fig. 4a–c). The calculated IC_50_ values were 27.83 ± 7.99 µg/ml for 48 h of NP incubation, 2.31 ± 0.32 µg/ml for 72 h of NP incubation, respectively 9.01 ± 4.68 µg/ml for 96 h of NP incubation. While IONP_CO_ showed nearly no effect on the metabolic activity of HeLa cells at 96 h compared to untreated controls, even at very high doses (200 µg/ml), incubation with IONP_DOX_ for 96 h showed a pronounced effect, showcasing a reduction in signal of 62.62 ± 2.05% (P = 0.002). All data were statistically significant compared to controls (non-treated cells), as shown by One-way ANOVA analysis. Moreover, Two-ways ANOVA statistical analysis showed a significant difference between IONP_CO_ and IONP_DOX_ (P < 0.0001 for 48 h; P < 0.0001 for 72 h; P < 0.0001 for 96 h). Also, the presence of DOX in the construct induced a significant cytotoxic effect in the HeLa cells (P = 0.0003 for 48 h; P < 0.0001 for 72 h; P < 0.0001 for 96 h).

**Figure 5:**
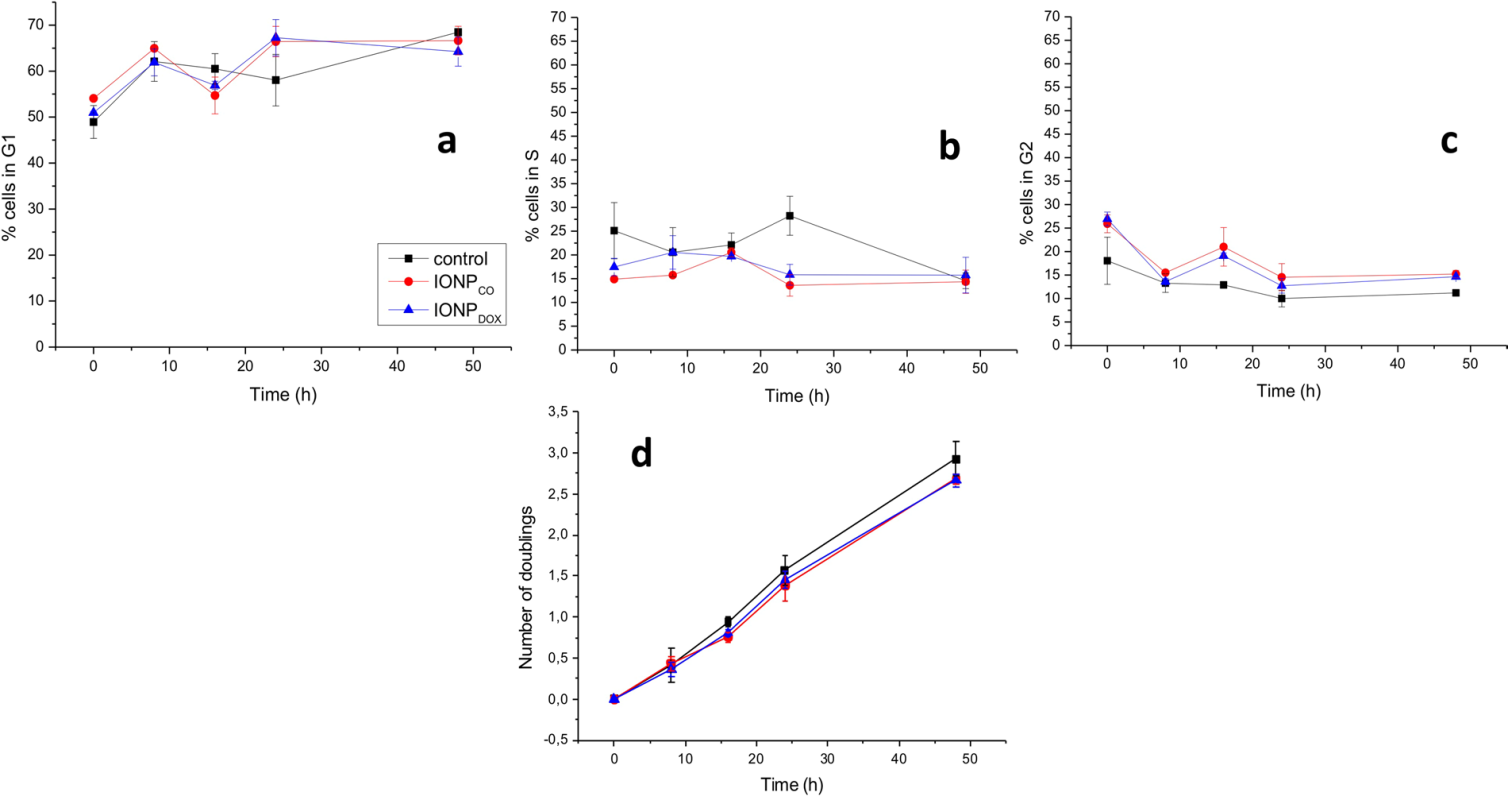
Flow cytometry of HeLa cells after IONP exposure during 16 h. Cell cycle distribution (**a–c**) and doubling time (**d**) of HeLa cells incubated with 0, respectively 100 μm IONP during 16 h (n = 3). Data are presented as percentage of untreated control and are shown as mean ± SEM (n = 3).

Incubation with IONP for 16 h caused a change in the cell cycle distribution (Fig. 5a–c), evidenced through an increase in the number of cells in G_2_ phase, compared to non-treated controls (Fig. 5c). This effect was observed at the time of removing the NP (25.90 ± 1.90 for IONP_CO_, 26.95 ± 1.45 for IONP_DOX_, respectively 18.03 ± 6.15 for control cells, where P = 0.05 for IONP_CO_, P = 0.03 for IONP_DOX_, compared to control), and again at 16 h after removing the NP (21.00 ± 4.10% for IONP_CO_, 19.10 ± 0.60% for IONP_DOX_ and 12.90 ± 0.70% for untreated controls; P = 0.013 for IONP_CO_, respectively P < 0.001 for IONP_DOX_ compared to control). The G_2_/M fraction decreased at 24 h which was paralleled by an increase of the fraction of G_1_ cells, suggesting induction and release of a temporary G_2_/M block. Measurements of cell division (number of cell doublings) (Fig. 5d) showed a minimal but statistically significant difference only at 16 h after nanoparticles removal caused by NPs (untreated controls vs IONP_CO_: P = 0.04; untreated controls vs IONP_DOX_: P = 0.03) as the effect was independent of DOX loading (IONP_CO_ vs IONP_DOX_: NS).

The clonogenic survival assay (Fig. 4d) emphasized the biocompatible character of the DOX-free NP with SF(IONP_CO_) = 1.07 ± 0.38, while the incorporation of DOX in the IONP polymeric shell caused a reduction in HeLa cells survival with SF(IONP_DOX_) = 0.56 ± 0.14 which was attributed to the release of DOX in the cells.

## Discussion

In this study we designed and synthesized polyethylene glycol (PEG)-functionalized iron oxide nanoparticles (IONP_CO_) for the encapsulation of the chemotherapeutic substance doxorubicin (IONP_DOX_). We determined the uptake efficacy of the IONP and evaluated their ability to induce cell death after DOX loading. Results showed that most of the loaded drug was released from IONP_DOX_ within 24 h, with a complete release at 70 h. Internalization of IONP_DOX_ and IONP_CO_ into HeLa cells occurred by pino- and endocytosis, with both IONPs accumulating in the peri-nuclear area. DOX-free nanoparticles proved to be biocompatible for HeLa cells, while the cells treatment with IONP_DOX_ determined a concentration and time-dependent decrease of cells proliferation.

The investigations highlight the intracellular fate of IONP after discontinuing the NP exposure, both quantitatively (through accurate measurements of the retained Fe_3_O_4_ per cell) and qualitatively (through electron microscopy imaging, in relation to the cellular compartments). Moreover, the study evaluates the IONP influence on the cell cycle and long-term proliferation/clonogenic survival after discontinuing the NP exposure.

IONP_DOX_ were made using a three-step synthesis method. The novel method that we have developed is adequate for large scale extension due the advantages like ease, low costs, high yield synthesis and reproducibility. In a first step, bare Fe_3_O_4_ nanoparticles were produced by modified room temperature chemical co-precipitation similar to^31^, resulting in highly crystalline face-centred spinel structured magnetite nanoparticles^32,33^ (Fig. 1d). Post-synthesis conjugation of the iron oxide cores using PEG (molecular weight 6 KDa) resulted in individual coverage of the IONP with a low crystalline organic phase, forming core-shell-like nano-constructs with high stability and positive surface charge (Fig. 1a–d). Previous results showed that PEG-conjugated nanoparticles have a positive charge in solutions with pH<8^34^, in concordance with our measurements. Dissociation of DOX ∙ HCl in water not only determined a change of IONP charge due to alteration of ions concentration in loading solution, but also led to higher hydrodynamic diameters, following DOX entrapment and interaction with PEG shells.

A challenge in developing nanoparticle-based drug delivery systems is finding an optimal design that enables internalizing, as well as retention in targeted cells. The microscopy investigations emphasized the nanoparticles localization in the cytoplasm of the cells (Fig. 2). In addition, a slight gradient effect of DOX, which might be a result of drug release from the IONP was observed (Fig. 2e and Supplementary Fig. S3). These results correlate with the DOX release data, considering the fact that the fluorescence microscopy investigations were done at 24 h after the end of NP treatment. Moreover, Fig. 2e proves the stability of DOX loading in IONP and suggests that the release is only triggered by the intracellular environment. In case of free DOX (Supplementary Fig. S3), due to its small size, directly diffused into the nucleus of the cell after few hours of incubation, while IONP_DOX_ samples were showing signal mainly in the cytoplasm and weak signal in the nuclei (Supplementary Fig. S3). Instability of DOX loading in IONP in stock solutions would cause a release of DOX in the buffer solution and thus the direct diffusion of the chemotherapeutic molecule in the cell nucleus accompanied by a lack of fluorescence signal from the cytoplasmic compartment.

Similar results were reported by Zhang Y. *et al*.^35^ for 180 nm Poly(ethylene glycol)- doxorubicin-curcumin nanoparticles which were mainly located in the vicinity of the nucleus at low incubation times, while the free drug diffused into the nucleus of the cells, due to a differentiation in the uptake pathways. This changed with time, as the active substances were released from the constructs.

For all experimental conditions, agglomerates of IONP could be observed both in the peri-nuclear area and the cytoplasm (Figs. 2, 3). The presence of IONP_CO_ was mostly observed in vesicle-like structures (Fig. 3a,b), due to entrapment in the endo-/lysosomal compartments^36^, while IONP_DOX_ were also found in the cytoplasm (Fig. 3c,d). Bypassing the endo-/lysosomal system is a requirement in the design of intracellular drug delivery systems^37^.

Petros *et al*.^38^ stated that nanoparticles having higher hydrodynamic diameter are transported across the cellular membrane via clathrin-mediated endocytosis or macropinocytosis. This was confirmed by Pearson *et al*.^39^ and Jana^40^. Bannunah *et al*.^41^ showed that dimension does not play an important role in the NP mechanism of internalizing, but it is dependent on surface charge; also, there is more than one mechanism involved in the internalizing of the same type of NP. Our results show that both types of constructs are internalized via macropinocytosis (Fig. 3e and Supplementary Fig. S4c) and sometimes smaller aggregates are internalized via endocytosis (Fig. 3f and Supplementary Fig. S4d). Both mechanisms were observed for DOX-loaded and DOX-free IONP, meaning that the cells do not use one mechanism or another based on surface charge, but rather on dimension. Extensions of the cellular membrane surround the NP agglomerates in the vicinity of the membrane, forming micrometre-sized vesicles and getting the constructs into the intracellular compartment. Eventually, these get trapped in lysosomes for cellular disposal (Fig. 3g and Supplementary Fig. S4e).

Our observations agree with results from other publications^26^. However, in case of DOX-loaded constructs, the major fraction of NP seem to escape the lysosomal trapping and to be freely located into the cytoplasm at 24 h after discontinuing the exposure of HeLa cells to IONP_DOX_ (Fig. 3c,d). The development and fate of PEG conjugated iron oxide nanoparticles to escape the endo-/ lysosomal trapping in the context of drug delivery is not well studied. This bypassing approach of iron oxide nanoparticles has been investigated for the purpose of radiosensitization, in case of dextran coated iron oxide nanoparticles conjugated with a cell penetrating peptide (TAT)^42^ and citrate coated superparamagnetic iron oxide nanoparticles^43^.

Lysosome function is to digest the internalized material taken up by the cell by means of endocytosis^44^, thus escaping or bypassing the lysosome uptake might be a solution to improve organelle specific targeting. In case of nanoparticle formulas, endosome and lysosome inclusions might also be an obstacle for effective treatment, as stated by Lloyd^45^. Whereas nano-carrier degradation by lysosome microenvironment and liberation of the active substance may still be considered one important principle of nanoparticle-based drug delivery systems, lysosome membrane can act as a natural barrier against efficient drug release^46^. In this case, cytosolic delivery of the drugs by nanoparticles escaping lysosomal entrapment^47,48^ might be a key to successful killing of the cancer cells.

Besides efficient uptake of nanoparticles in cancer cells, their retention is also important so that an effective high concentration can be reached. While small nanoparticles (diameters lower than 50 nm) undergo exocytosis within 24 h of uptake, larger nanoparticles or aggregates are retained for longer periods of time^49^. Our microscopy investigations emphasized the presence of IONP after 40 h (16 h of incubation with NPs and additional 24 h incubation without NPs), while quantitative measurements showed that, at this time-point, a 3.6-fold higher concentration of Fe_3_O_4_ was measured in IONP_DOX_ compared to IONP_CO_ (Fig. 3h). This difference in internalized Fe_3_O_4_ in the two groups of IONP-exposed HeLa cells might be due to the difference in hydrodynamic diameter (almost double for IONP_DOX_ compared to IONP_CO_), but also due to the induction of cell death in case of DOX-loaded nanoparticles, which can artificially increase the concentration Fe_3_O_4_/cell. To the best of our knowledge this is the first study to evaluate the intracellular retaining and fate of PEG-coated iron oxide nanoparticles (qualitatively, in relation to the cellular compartments and also quantitatively, by providing an accurate concentration of NP per individual cell) at periods of time longer than one complete cell cycle, after the termination of NP exposure.

Considering compatibility to human blood, our results showed biocompatibility for both IONP_CO_ and IONP_DOX_ (Supplementary Material Section 7), matching data from the literature^50–52^. Concerning the cytotoxicity and proliferation exhibited by HeLa cells after IONP exposure, our data (Fig. 4a–c) showed that, in the first days of interaction between cells and PEG-functionalized NP, a weak cytotoxic effect occurred already at very small concentrations, which did not increase with concentration, after a certain threshold, the amount of cytotoxicity being maintained almost constant. Similar results were obtained by Xia *et al*.^53^ for a redox responsive polyethylene glycol-Fe_3_O_4_ nanoparticles self-assembled micelles. However, at later time points (96 h), the cell proliferation was maintained above 80% limit (relative to control), which is a threshold for biocompatibility (ISO 10993-12:2001(E)^54^). Long-term monitoring (Fig. 4d) showed that IONP_CO_ did not affect the clonogenic survival of HeLa cells after 14 days. Other studies also reported PEG-coated iron oxide nanoparticles as biocompatible^55–57^. Feng *et al*.^26^ showed that such nanoparticles could induce autophagy *in vitro*, but otherwise showed no obvious signs of *in vivo* toxicity in BALB/c mice.

Results for IONP_DOX_ showed that DOX-containing NP caused significant cytotoxicity and growth inhibition in HeLa cells compared to untreated control samples (Fig. 4a–c). This effect was concentration and time dependent, showing that these constructs can be efficiently used to induce cell death in human cervical adenocarcinoma cells. The lack of recovery at 96 h (as compared to IONP_CO_) suggests that IONP_DOX_ not only caused growth delay but also real cytotoxicity. The clonogenic survival data confirmed this by showing a reduced SF in cells treated with IONP_DOX_ after 14 days compared to IONP_CO_-treated cells (Fig. 4d).

The cell cycle arrest at 16 h after IONP_CO_ treatment was also observed for IONP_DOX_ (Fig. 5) indicating little effect of DOX released from the nanoparticles. In all, results showed no statistically significant difference in cell cycle distribution between the IONP_CO_ and IONP_DOX_ groups (Fig. 5).

This study shows the generation and characterization of polyethylene glycol-functionalized iron oxide nanoparticle that have shown efficient internalizing and retention in human cervical adenocarcinoma cells. Highly crystalline, bio- and hemocompatible nanoparticles, IONP have the ability to encapsulate and deliver the chemotherapeutic doxorubicin directly into the intracellular cytoplasmic compartment and thereby efficiently causing cell death in the cells. This makes them potential candidates for nanoparticle-mediated and chemotherapy-induced inactivation of tumour cells.

## Methods

### Synthesis and characterization of the IONP

The synthesis of the IONP was done in 3 steps (described in more detail in the Supplementary Material Section 1): (1) Synthesis of bare iron oxide nanoparticles through chemical co-precipitation, (2) post-synthesis functionalizing of iron oxide nanoparticles with polyethylene glycol (PEG) 6000 Da (IONP_CO_) and (3) optional loading of the IONP_CO_ with doxorubicin (only in IONP_DOX_).

The morphology, crystallinity and mineralogical composition of the IONP was examined using transmission electron microscopy (TEM) on a Tecnai G2 F30 S-TWIN HR-TEM (Thermo Fisher Scientific, Hillsboro, OR, USA) equipment with selected area electron diffraction (SAED) module, for which sample preparation has been described previously^31^. The analysis of the loading efficiency of DOX into the nano-constructs is described in Supplementary Material Section 2.

The hydrodynamic diameter and surface charge (zeta potential) of IONP were characterized using a Delsa Nano C instrument (Beckman Coulter, Brea, CA, USA) and recorded using the DelsaNano 3.73 software (Beckman Coulter). The measurements were done for freshly prepared nanoparticle suspensions in ultrapure water without prior ultrasound dispersion, based on the existing international documentary standards ISO 13321:1996^58^ and ISO 22412: 2008b^59^.

The release kinetics of DOX from IONP_DOX_ was measured for media with different biologically relevant pH: 7.4, 6.5 and 4.7. Studies were done in standard conditions of temperature and humidity (37 ± 2 °C, 5 ± 1% CO_2_). Samples were prepared and analysed as described in the Supplementary Material Section 3 and performed once in triplicate.

### Cell culture

The biological evaluation of the IONP was performed on the human cervical adenocarcinoma cell line HeLa, which was obtained from the Tumour Cell Bank of the German Cancer Research Centre (DKFZ, Heidelberg, Germany). This cell line was chosen because it has been previously used in a variety of studies^49,60,61^ evaluating novel nanoconstructs and thereby allows a comparison of results. Cells were cultured in Dulbecco’s modified Eagle’s medium (DMEM) (Biochrom, Merck KGaA, Darmstadt, Germany) supplemented with 10% FBS (Biochrom, Merck KGaA). Cell cultures were maintained at 37 °C in a humidified incubator (95% air, 5% CO_2_).

### Treatment and incubation with IONP

HeLa cells were seeded in appropriate concentration for each investigation and allowed to attach for 4 h. Afterwards, the culture medium was replaced with fresh medium containing nanoparticles in the respective concentrations and incubated for 16 h.

### Uptake and retention of IONP

The uptake and retention of IONP by HeLa cells was evaluated by three microscopic methods generating complementary information on the localizing of the nanoparticles in relation to the cultured tumour cells. The preparation of the samples for microscopy is described in the Supplementary Material Section 4. Optical visualization of IONP in HeLa cells 24 h after incubation (for 16 h) in presence of nanoparticles was performed using a Prussian Blue staining, resulting in a light blue colouring of sub-micron structures. Fluorescence imaging was possible due to native property of doxorubicin. Optical and fluorescence microscopy were performed using a Leica DMRE microscopy equipped with a Leica DFC3000G camera (Leica Mikrosysteme Vertrieb GmbH Mikroskopie und Histologie, Wetzlar, Germany) and an Axio Observer Z1 microscope (ZEISS, Oberkochen, Germany) equipped with an Axiocam 506 camera. Images were acquired using ZEN 2 software (ZEISS). Samples for transmission electron imaging were prepared similarly as for optical and fluorescence microscopy imaging (as described in Supplementary Material Section 4). Images were acquired using a Zeiss EM 10 transmission electron microscope (ZEISS) equipped with an Olympus Megaview G2 camera (Olympus Europa SE & Co. KG, Hamburg, Germany).

Particle-induced X-Ray emission (PIXE) was performed using a 3 MV Tandetron accelerator with a 2.7 MeV proton beam and an in-air irradiation setup^62^. The characteristic X-Rays were recorded by an Amptek silicon drift detector (SDD) positioned at 45° with respect to the beam direction and the spectra were processed with the GUPIXWIN 2.2.4 software^63^. The SDD resolution is 130 eV at 5.9 keV (K_α_ line of ^55^Mn). The concentration of Fe_3_O_4_ per cell was calculated by normalizing the output values to the viable cell number at each corresponding time point.

### Proliferation and clonogenic survival

The quantitative effects of the IONP on proliferation kinetics were evaluated using a tetrazolium salt-based proliferation assay (MTT, Sigma-Aldrich Chemie GmbH, Taufkirchen, Germany). Dose-response curves were obtained for concentrations up to 200 μg/mL IONP continuous exposure for 48, 72 and 96 h. Samples were prepared as described in the Supplementary Material Section 5.

Cells for longer term survival evaluation (14 days) were incubated with IONP for 16 h and then detached and reseeded into the colony formation assay at 200 cells/ 25 cm^2^ flask as described in Supplementary Material Section 5.

The surviving fraction (SF) was fitted with the linear-quadratic model (ln(SF) = -(αD + βD^2^)) using the non-linear regression tool of SigmaPlot 12 (Systat Software GmbH, Erkrath, Germany)^49^.

### Cell cycle distribution and doubling time

The change in cell cycle distribution and division of HeLa cells exposed during 16 h with IONP was evaluated for cells treated and stained as described in Supplementary Material Section 6. Acquisition was performed on the BD FACSLyric (BD Biosciences, San Jose, CA, USA), and analysed using FlowJo 10.5 software (BD Biosciences).

### Statistical analysis

All data are presented as mean ± SEM from three independent experiments, unless specified otherwise. Statistical analysis was done using t-test, one-way ANOVA (SigmaPlot 12) and two-way ANOVA (Prism 5, GraphPad, San Diego, USA).

## Supplementary information

Supplementary information.

## Data Availability

All data generated or analysed during this study are included in this published article (and its Supplementary Information File).
