# Supplementary material for: Efficient uptake and retention of iron oxide-based nanoparticles in HeLa cells leads to an effective intracellular delivery of doxorubicin
Source: Sci Rep. 2020 Jun 29;10:10530. doi: 10.1038/s41598-020-67207-y (PMC7324358; doi:10.1038/s41598-020-67207-y)
Supplement: Supplementary file 1 — Supplementary information. [file 41598_2020_67207_MOESM1_ESM.doc]

**Supplementary information**

**Efficient uptake and retention of iron oxide-based nanoparticles in HeLa cells leads to an effective intracellular delivery of doxorubicin**

R.C. Popescu^1,2,3^, D. Savu^1*^, I. Dorobantu^1^, B.S. Vasile^2^, H. Hosser^4^, A. Boldeiu^5^, M. Temelie^1^, M. Straticiuc^6^, D.A. Iancu^6^, E. Andronescu^2^, F. Wenz^7^, F. Giordano^3^, C. Herskind^3^, M.R. Veldwijk^3*^

^1^“Horia Hulubei” National Institute for Research and Development in Physics and Nuclear Engineering, Department of Life and Environmental Physics, Reactorului 30, 077125, Magurele, Romania

^2^Politehnica University of Bucharest, Department of Science and Engineering of Oxide Materials and Nanomaterials, Polizu 1-7, 011061, Bucharest, Romania

^3^Heidelberg University, Medical Faculty Mannheim, Universitätsmedizin Mannheim, Department of Radiation Oncology, Theodor-Kutzer-Ufer 1-3, 68167, Mannheim, Germany

^4^Heidelberg University, Medical Faculty Mannheim, Universitätsmedizin Mannheim, Center for Biomedicine and Medical Technology, Department of Anatomy and Developmental Biology, Theodor-Kutzer-Ufer 1-3, 68167, Mannheim, Germany

^5^National Institute for Research and Development in Microtechnologies, Laboratory of Nanobiotechnology, Erou Iancu Nicolae 12A, 077190, Bucharest, Romania.

^6^”Horia Hulubei” National Institute for Research and Development in Physics and Nuclear Engineering, Department of Applied Nuclear Physics, Reactorului 30, 077125, Magurele, Romania.

^7^University Medical Center Freiburg, Hugstetter Straße 55, 79106 Freiburg, Germany

1. **Production of IONP**

For the synthesis of bare Fe_3_O_4_ nanoparticles, a modified chemical co-precipitation method was used by adding dropwise a solution of ferric (FeCl_3_, Sigma-Aldrich Chemie GmbH, Taufkirchen, Germany) and ferrous (FeSO_4_·7H_2_O, Sigma-Aldrich Chemie GmbH) ions (ratio of 0.625) into a basic solution, under magnetic stirring. The resulting colloids were washed several times with ultrapure water by magnetic separation and the nanoparticles were dried at 50^o^C overnight. The nanoparticle powder was suspended in a 10:1 ethanol:water solution by ultrasound dispersion. Polyethylene glycol 6000 Da (Sigma-Aldrich Chemie GmbH) was added into the nanoparticles solution at a 1:1 ratio and the resulted medium was heated, under magnetic stirring. Afterwards, the resulted IONP_CO_ were washed several times with ultrapure water and suspended in ultrapure water. Doxorubicin hydrochloride (Cayman Chemical Company, Michigan, USA) was added to half of the IONP (138 μM DOX) and let for 24 h under magnetic stirring, at room temperature, in the dark. After this time, the nanoparticles were magnetically separated from the medium and the remaining solution was kept for measuring the loading ability of DOX into the nanoparticles. The resulted nanoparticles were washed 3 times with ultrapure water and the remaining medium was kept for loading measurements. At the end, the IONP_DOX_ were suspended in ultrapure water.

1. **Quantitative determination of DOX loading efficiency in IONP_DOX_**

In order to determine the loading efficiency of DOX into the nano-constructs, a calibration curve (absorbance depending on concentration) was done by making serial dilutions of DOX in ultrapure water. UV-VIS spectrophotometric measurements were done for the medium resulted after the loading of the drug. The measurements were done at 480 nm ([[1]](#endnote-2)) using Sunrise Mithras LB 940 spectrophotometer (Berthold Technologies GmbH, Bad Wildbad, Germany) and the absorbance values were intercalated into the calibration graphic (Table S1). The loading efficiency was calculated according to Kamba et al ([[2]](#endnote-3)), considering the difference between the total weight of the added drug and the weight of the non-encapsulated drug (in the washing water), normalized to the total weight of nanoparticles. Thus, the total weight of the added drug was 2.5 mg and the weight of the non-encapsulated drug was 1.39 mg, yielding 1.11 mg encapsulated DOX. The total weight of nanoparticles was 100 mg, resulting in 1.11 wt% DOX.

**Table S1:** Dependence of absorbance on DOX concentration in water and DOX-loading efficiency measurements for DOX-IONPs;

**Calibration curve** **Measurement in the loading water**

| DOX conc (μg/ml) | Absorbance  ____________________________ | | | | mean value |  | absorbance from rest of water | concentration of DOX in the water |
| --- | --- | --- | --- | --- | --- | --- | --- | --- |
| 1.953125 | 0.072 | 0.063 | 0.063 | 0.065 | 0.066 |  | 0.498 0.495 0.496 | 55.682 |
| 3.90625 | 0.111 | 0.086 | 0.086 | 0.086 | 0.092 |  |  |  |
| 7.8125 | 0.145 | 0.127 | 0.125 | 0.130 | 0.132 |  |  |  |
| 15.625 | 0.214 | 0.203 | 0.197 | 0.201 | 0.204 |  |  |  |
| 31.25 | 0.337 | 0.336 | 0.327 | 0.290 | 0.323 |  |  |  |
| 62.5 | 0.555 | 0.566 | 0.566 | 0.541 | 0.557 |  |  |  |
| 125 | 0.924 | 0.969 | 0.940 | 0.959 | 0.948 |  |  |  |
| 250 | 1.688 | 1.658 | 1.685 | 1.440 | 1.618 |  |  |  |
| 500 | 2.429 | 2.493 | 2.488 | 0.365 | 1.944 |  |  |  |
| 1000 | 2.632 | 2.629 | 2.618 | 0.891 | 2.193 |  |  |  |
|  |  |  |  |  |  |  |  |  |

Concentration of non- encapsulated DOX (in 25 mL total volume) = 55.7 μg/mL

Total weight of non-encapsulated DOX = 1.39 mg

Total weight of drug (added at beginning) = 2.5 mg/mL

Concentration of nanoparticles = 4000 μg/mL

Total weight of nanoparticles = 100 mg


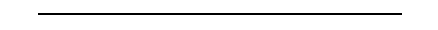
 = 1.11 wt%

1. **Release kinetics measurements**

The release kinetics of the chemotherapeutic substance from IONP_DOX_ was measured for different relevant mediums: (1) physiological plasma, pH=7.4 (Phosphate Buffer Saline- PBS ([[3]](#endnote-4))), (2) tumor microenvironment, pH=6.5 (acid PBS, pH adjusted using 1M HCl) and (3) endosome microenvironment (Endosome-like buffer, obtained using 12 mM NaH_2_PO_4_, 145 mM NaCl, 4.7 mM KCl and pH adjusted at 4.8 using Na_2_HPO_4_ solution). Suspensions of dried nanoparticles were done in each buffer at the concentration of stock solutions and incubated for different time intervals in standard conditions of temperature and humidity (37 ± 2 °C, 5 ± 1% CO_2_, more than 90% humidity). At each time-point, the buffers were separated from the IONP_DOX_ and kept for fluorescence measurements at 490 nm excitation and 593 nm emission wavelengths ([[4]](#endnote-5)), using the Mithras LB 940 spectrophotometer (Berthold Technologies GmbH, Bad Wildbad, Germany).

1. **Internalization of IONP in HeLa cells**

In order to evaluate the uptake and retention of IONP in HeLa cells, both ***optical and fluorescence microscopy*** imaging were employed. For this, 10^5^ cells/ well were seeded in 24- well plates and incubated during 4h; after this time, the culture medium was replaced with fresh medium containing nanoparticles (concentrations of 100 μg/mL of IONP_CO_, IONP_DOX_ or 1.11 μg/mL equivalent concentration of free DOX). Cells were incubated for additional 16h, then washed 3 times with PBS, detached and centrifuged (1700 rpm, 5 min), to completely eliminate the nanoparticles that were not in direct interaction with the cells. Afterwards, the supernatant was replaced with nanoparticle-free fresh culture medium, cells were seeded onto 10 mm round coverslips at a density of 5x10^4^ cells/ slide and incubated for additional 24 h. The cells were fixed with 3.7% paraformaldehyde (PFA) in PBS during 10 minutes. Samples prepared for optical microscopy investigations were stained with Prussian Blue (Sigma-Aldrich Chemie GmbH) (2% in PBS : 1M HCl, ratio 1:1, for 10 minutes, at 37^o^C) ([[5]](#endnote-6)). The slides were mounted with DAPI-containing mounting medium (Vectashield, BIOZOL Diagnostica Vertrieb GmbH, Eching, Germany), at room temperature and microscopy investigations were done using a Leica DMRE microscope equipped with a Leica DFC3000G camera (Leica Mikrosysteme Vertrieb GmbH Mikroskopie und Histologie, Wetzlar, Germany).


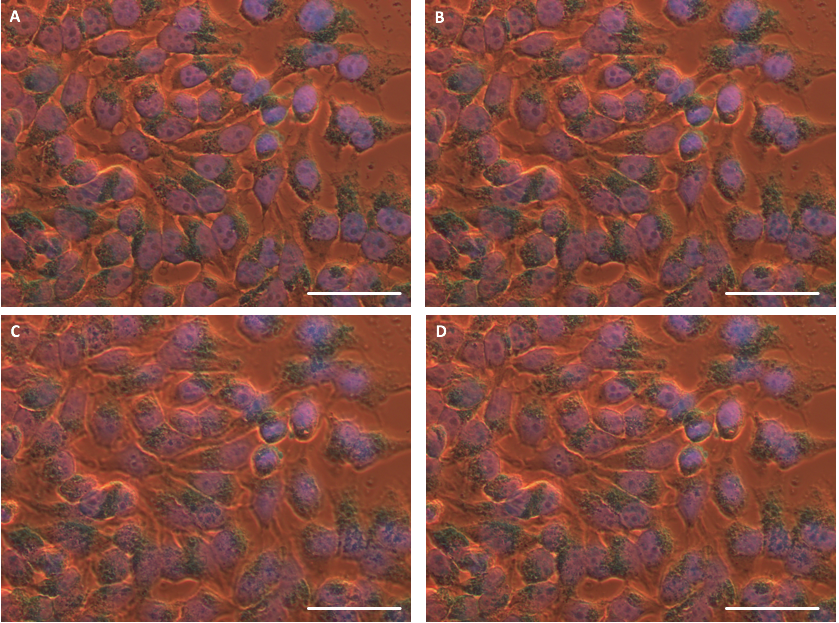


**Figure S1:** HeLa cells exposed for 16 h to 100 μg/mL IONP_CO_; phase contrast and fluorescence overlay, Prussian Blue and DAPI staining, different focusing planes; Scale = 50 μm.


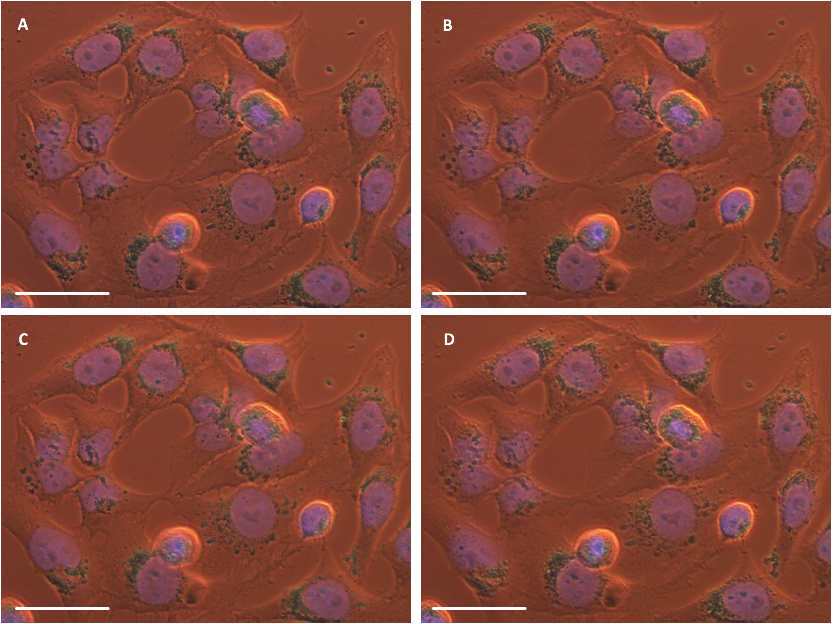


**Figure S2:** HeLa cells exposed for 16 h to 100 μg/mL IONP_DOX_; phase contrast and fluorescence overlay, Prussian Blue and DAPI staining, different focusing planes; Scale = 50 μm.


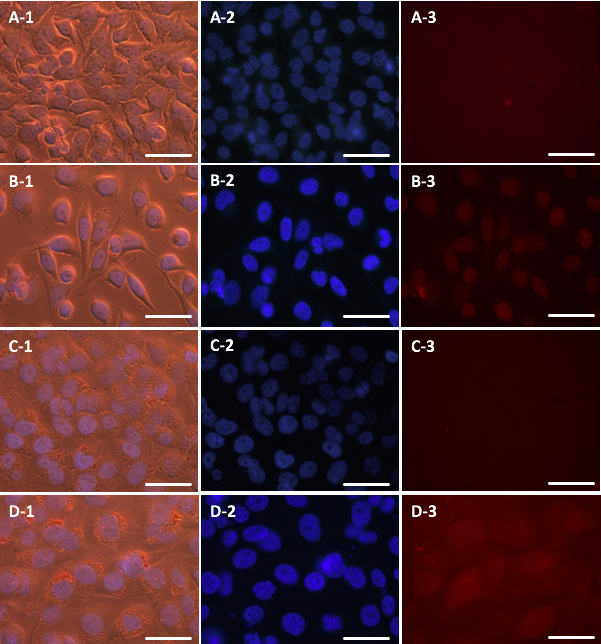


**Figure S3:** Morphological characterization of HeLa cells after IONP/ DOX treatment. HeLa cells exposed for 16 h to: (A 1-3) control; (B 1-3) DOX equivalent concentration of 100 μg/mL IONP (= 1.11 μg/mL); (C 1-3) 100 μg/mL IONP_CO_; (D 1-3) 100 μg/mL IONP_DOX_; 1- phase contrast image with DAPI staining; 2- DAPI staining; 3- DOX (TRITC); Scale 50 μm.

Samples for ***transmission electron imaging*** were prepared by seeding 2x10^5^ cells/well in a 6 well plate, following with the same protocol as for optical and fluorescence microscopy imaging. After incubation, cells were washed 3 times with PBS and fixed with 3.7% PFA during 24h, then gently detached using a cell scraper. The samples were collected in 1.5 mL tubes (Eppendorf, Hamburg, Germany) and washed 3 times with PBS by centrifugation (1000 xg, 5 min). Afterwards, cells were additionally fixed with 2% OsO_4_ (Plano GmbH, Wetzlar, Germany) during 1h at room temperature and then dehydrated using solutions of ethanol and acetone with increasing concentration (Carl Roth GmbH&Co. KG, Karlsruhe, Germany); samples were embedded in Epon (SERVA Electrophoresis GmbH, Heidelberg, Germany) and cut into 90 nm slices, which were placed onto copper or nickel grids (Plano GmbH); counterstaining was done with uranyl acetate (SERVA Electrophoresis GmbH) or lead citrate (Merck KGaA, Darmstadt, Germany); images were acquired using a Zeiss EM 10 transmission electron microscope (ZEISS, Oberkochen, Germany), equipped with an Olympus Megaview G2 camera (Olympus Europa SE & Co. KG, Hamburg, Germany).


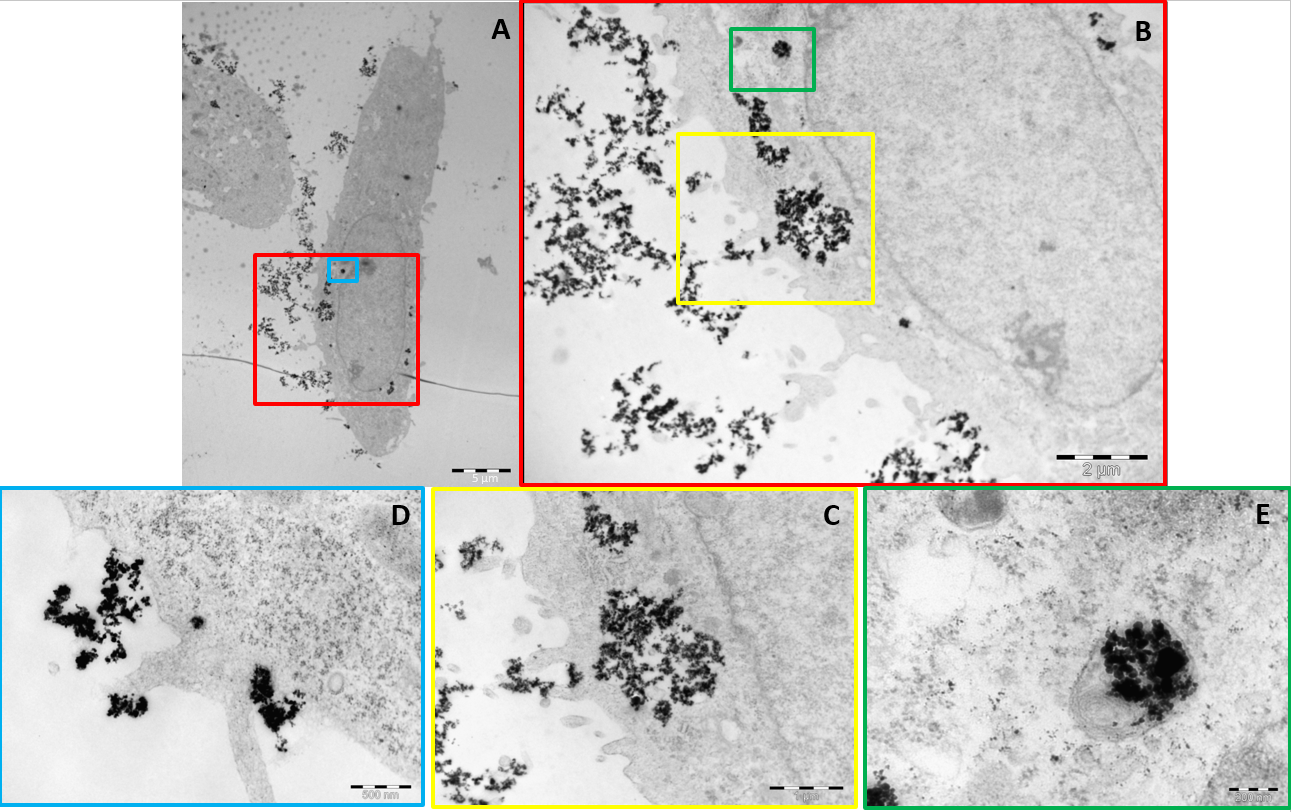


**Figure S4:** Internalization of IONP in HeLa cells. HeLa cells exposed to 100 μg/mL IONP for 16h: (A) overview of the whole cell, scale = 5 μm; (B) magnification of the area marked with red square in (A), scale = 2 μm, image rotated 20^o^; (C) magnification of the area marked with yellow square in (B), scale = 500 nm; (D) magnification of the area marked with blue square in (A), scale = 1 μm and (E) magnification of the area marked with orange square in (B), scale = 200 nm.

1. **Proliferation and surviving fraction**

The quantitative proliferation kinetics of the IONP was evaluated by an MTT (Sigma-Aldrich Chemie GmbH) tetrazolium salt-based assay, to obtain dose-response curves for concentrations up to 200 μg/mL. Measurements were performed at 48, 72 and 96 h, while different cell numbers were seeded in order to avoid confluence (48 and 72 h: 3x10^3^ cells/well; 96h: 1.5x10^3^ cells/well). Corresponding cell concentrations were seeded in 96 well plates and incubated for 4 h in standard conditions of temperature and humidity, to allow the attachment of the cells. Meanwhile, equivalent concentrations of DOX- loaded and DOX-free IONPs were made using binary serial dilutions in complete DMEM by ultrasound dispersion and replaced the existing medium in the wells. At the end of the incubation time with IONP (48, 72, respectively 96h), 10 μL of 5 mg/mL MTT in PBS solution were added to each well and incubated for additional 2h; DMSO was used to solubilize the formazan crystals in each sample, while the absorbance was measured at 570 nm using a Tecan infinite M200 microplate reader (Tecan Group Ltd., Männedorf, Switzerland). Viability of each sample was calculated as reported to controls (untreated cells); blank samples (wells with equivalent (DOX-) IONP concentrations but no cells) were prepared in order to eliminate possible interferences.

Samples for Colony formation assay (CFA) were prepared as following: 10^5^ cells/ well were seeded in 24 well-plates and incubated in standard conditions for 4h, to allow attachment. The culture medium was then replaced with 100 μg/mL IONP containing fresh medium and cells were incubated for additional 16 h. After this time, the cells were carefully washed 3 times with PBS. Cells were detached and different dilutions of each sample were made (40-1000 cells/ 1000 μL complete culture medium). Cells were seeded at different densities in T25 cell culture flasks (TPP Techno Plastic Products AG, Trasadingen, Switzerland) with 5 mL complete culture medium and incubated for 14 days at 37 °C and 95% humidity. After this time, samples were fixed using a methanol/ acetic acid solution and stained using crystal violet as described ([[6]](#endnote-7)); counting and scoring was done as described in ([[7]](#endnote-8)). The surviving fraction (SF) was fitted with the linear-quadratic model (ln(SF)= -(αD+βD^2^)) using the non-linear regression tool of SigmaPlot 12 (Systat Software GmbH, Erkrath, Germany) (^7^)

1. **Cell cycle distribution and doubling time**

7x10^5^ cells were seeded in 25 cm^3^ flasks and incubated with 10 μM bromodeoxyuridine (BrdU, Sigma-Aldrich Chemie GmbH) for 20h before the first measurement time point. After first 4h of incubation, medium was replaced with fresh medium with NPs and BrdU and incubated for additional 16h. Afterwards, samples were washed, detached and fixed at 0, 4, 12, 24 and 48 h after irradiation, using a protocol as in ([[8]](#endnote-9)). BrdU-labeled cells were fixed in 70% ice-cold ethanol at -20℃ for at least 24h. Cells were treated with 2 mol/L HCl /0.5% Triton X-100 for 30 min, then washed in PBS/0.5% BSA and incubated in 0.1 mol/L sodium tetraborate for 2 minutes. After an additional wash in PBS/0.5% BSA, cells incubated with monoclonal anti-BrdU antibody (1:300; clone 3D4; BD Biosciences, San Jose, CA, USA) for 30 min. Cells were washed in PBS/0.5% BSA and then incubated with a FITC-conjugated second antibody for 30 min (1:300; AP181F, Merck KGaA). Finally, cells were stained with 5 µg/ml propidium iodide and 50 µg/ml RNase in PBS for 30 min. Acquisition was performed on a BD FACSLyric (BD Biosciences), and analyzed using FlowJo 10.5 software (BD Biosciences).

1. **Hemocompatibility of IONP**

The hemocompatibility of IONP was assessed according to ASTM standard E2524-08 (Standard test method for analysis of hemolytic properties of nanoparticles), method based on determining the released hemoglobin into plasma free hemoglobin (PFH) as a percentage of the total blood hemoglobin (TBH) after the exposure of the blood to IONP.

The study was approved by the National Institute of Physics and Nuclear Engineering’s Ethics Committee (Approval No. 58/29.07.2019) and was carried out in accordance with the guidelines and regulations stated in the Declaration of Helsinki. The informed consent was obtained from all the subjects involved in the study.

Heparinized whole blood samples from 3 human healthy donors were used. The three samples were pooled in equal amount. A fraction of the blood was centrifuged, and supernatant (plasma) was collected. Remaining blood was diluted 10 times in Ca/Mg free Dulbecco’s PBS (DPBS, Thermo Fisher Scientific wissenschaftliche Geräte GmbH, Vienna, Austria). Drabkin’s reagent (cyan methemoglobin reagent, Sigma-Aldrich Chemie GmbH) was used to determine PFH and TBH of blood sample. Diluted blood and plasma were mixed with Drabkin’s reagent and hemoglobin standard were prepared in Drabkin’s reagent in a concentration range of 0.025-0.8 mg/mL. Quality control standards of 0.0625 mg/ml, 0.125 mg/mL and 0.625 mg/mL are also prepared and used to assess the correctitude of the assay. Absorbance at 540 nm was measured. Appropriate dilution factors were used. Based on calculated concentration blood was adjusted to 10±1 mg/mL with DPBS.

The nanoparticle samples were prepared in DPBS and serial dilutions (1:5) were made in DPBS, in order to obtain the final concentrations of 100, 20 and 4 μg/mL IONP. Positive control was 1% Triton X- 100 (Sigma-Aldrich Chemie GmbH) in distilled water and respectively negative control PEG8000 (Sigma-Aldrich Chemie GmbH) and blank (DPBS).

100µl of sample of each concentration, positive and negative control were placed in microtubes. 700µl DPBS and 100µl diluted blood were added to each tube. Similar no-blood controls were prepared by replacing blood with DPBS in order to be able to subtract the absorbance given by particles that may remain in the supernatant and could give a false-positive reaction. All prepared samples were incubated for 3h at 37^o^C, by mixing the tubes every 30 min. Afterwards the tubes were centrifuged in order to remove IONP and intact red blood cells. Visual observation of each tube was done in order to eliminate potential samples with false-negative reaction that may happen if the nanoparticles adsorb the free hemoglobin released upon erythrocyte lysis [[[9]](#endnote-10)]. In this case the pellet will look loose or will present an intermediate phase of pale-red just above the pellet. All the IONP samples tested here and negative control has a similar appearance as a dense, compact red pellet with a homogenous, clear supernatant, in concordance to our previous tests on similar IONP [[[10]](#endnote-11)]

100µl of the supernatants were mixed in 1:1 ratio with Drabkin’s reagent. Absorbance of samples and controls, diluted blood, hemoglobin standards prepared as described above and bank Drabin’s reagent was measured at 540 nm in volumes of 200µl, using a plate reader (background correction applied- i.e. IONP in DPBS with no blood). All samples were worked in triplicate. Hemoglobin concentration was calculated based on standard curve constructed by hemoglobin standards absorbance and using appropriate correction given the dilution factors.

The hemolysis was calculated as following:

% hemolysis = PFH_sample_/ TBH_diluted_ x 100 (1)

Both IONP_CO_ and IONP_DOX_ did not produce any significant hemolytic effect at none of the concentrations analyzed, as the measured hemolytic potential (table S2) was below 5% (ASTM standard E2524-08).

**Table S2:** Hemolytic potential of IONPs;

| **NPs concentration (μg/mL)** | **Hemolytic potential (%)** | |
| --- | --- | --- |
|  | **IONP_CO_** | **IONP_DOX_** |
| 100 | 0.17±0.14 | -0.07±0.08 |
| 20 | 0.23±0.05 | 0.03±0.1 |
| 4 | 0.15±0.04 | -0,11±0.07 |

1. **References:**

1. Motlagh NSH, et al Fluorescence properties of several chemotherapy drugs: doxorubicin, paclitaxel and bleomycin. *Biomed Optics Express*. **7(6)**, 2400-2406 (2016) [↑](#endnote-ref-2)
2. # Kamba S.A. et al, ***In Vitro***Delivery and Controlled Release of Doxorubicin for Targeting Osteosarcoma Bone Cancer. *Molecules*, 18, 10580-10598 (2013).

   [↑](#endnote-ref-3)
3. <https://www.fda.gov/food/laboratory-methods-food/bam-r59-phosphate-buffered-saline-pbs-ph-74> (2019). [↑](#endnote-ref-4)
4. ## [Liang](https://pubs.rsc.org/en/results?searchtext=Author%3AJunyu Liang), J., et al, Simple and rapid monitoring of doxorubicin using streptavidin-modified microparticle-based time-resolved fluorescence immunoassay, ***RSC Adv*.**, **8**, 15621-15631 (2018).

   [↑](#endnote-ref-5)
5. Schlorf, T. et al. Biological Properties of Iron Oxide Nanoparticles for Cellular and Molecular Magnetic Resonance Imaging. *Int. J. Mol. Sci.*  **12**, 12-23 (2011). [↑](#endnote-ref-6)
6. Liu Q, Schneider F, Ma L, Wenz F, Herskind C. Relative biologic effectiveness (RBE) of 50 kV x-rays measured in a phantom for intraoperative tumor-bed irradiation. *Int J Radiat Oncol Biol Phys* **85**, 1127-33 (2013). [↑](#endnote-ref-7)
7. Burger N,et al.. A method for the efficient cellular uptake and retention of small modified gold nanoparticles for the radiosensitization of cells. *Nanomed* **10(6)**, 1365-73 (2014). [↑](#endnote-ref-8)
8. Nicoletti, I. Pagliacci, M. C., Grignani, F., Migliorati, G., Riccardi C. A rapid and simple method for measuring thymocyte apoptosis by propidium iodide staining and flow cytometry. *J.Immun Met*, **139(2)**, 271-9 (2019). [↑](#endnote-ref-9)
9. Neun, B.W., Ilinskaya A.N., Dobrovolskaia M.A. Updated method for In Vitro Analysis of Nanoparticle Hemolytic Properties in: *Characterization of Nanoparticles Intended for Drug Delivery. Methods in Molecular Biology*. (ed. McNeil S.), 1682 (Humana Press, 2018). [↑](#endnote-ref-10)
10. Temelie M., Popescu R.C., Cocioaba D., Vasile B.S., Savu D. Biocompatibility study of magnetite nanoparticle synthesized using a green method. *Romanian J. Phys.* **63**, 703 (2018). [↑](#endnote-ref-11)
